# Exploring barriers and facilitators of behavioural changes in dietary intake and physical activity: a qualitative study in older adults undergoing transcatheter aortic valve implantation

**DOI:** 10.1007/s41999-023-00774-1

**Published:** 2023-04-01

**Authors:** Dennis van Erck, Christine D. Dolman, José P. Henriques, Josje D. Schoufour, Ronak Delewi, Wilma J. M. Scholte op Reimer, Marjolein Snaterse

**Affiliations:** 1 Department of Cardiology, Amsterdam UMC, University of Amsterdam, Amsterdam, The Netherlands; 2 Department of Cardiothoracic Surgery, Amsterdam UMC, University of Amsterdam, Amsterdam, The Netherlands; 3 Faculty Health, Center of Expertise Urban Vitality, Amsterdam University of Applied Sciences, Amsterdam, The Netherlands; 4 Faculty of Sports and Nutrition, Center of Expertise Urban Vitality, Amsterdam University of Applied Sciences, Amsterdam, The Netherlands; 5 Research Group Chronic Diseases, HU University of Applied Sciences Utrecht, Utrecht, The Netherlands

**Keywords:** Dietary intake, Physical activity, Cardiac patients, Behaviour change, Patient perspectives

## Abstract

**Purpose:**

The majority of older patients, scheduled for a cardiac procedure, do not adhere to international dietary intake and physical activity guidelines. The purpose of this study was to explore barriers and facilitators regarding dietary intake and physical activity behaviour change in older patients undergoing transcatheter aortic valve implantation (TAVI).

**Methods:**

We conducted a qualitative study using semi-structured interviews with patients undergoing TAVI. Interviews were analysed by two independent researchers using thematic analysis, the capability, opportunity and motivation behaviour model was used as a framework.

**Results:**

The study included 13 patients (82 ± 6 years old, 6 females) until data saturation was reached. Six themes were identified, which were all applicable to both dietary intake and physical activity. Three following themes were identified as barriers: (1) low physical capability, (2) healthy dietary intake and physical activity are not a priority at an older age and (3) ingrained habits and preferences. Three following themes were identified as facilitators: (1) knowledge that dietary intake and physical activity are important for maintaining health, (2) norms set by family, friends and caregivers and (3) support from the social environment.

**Conclusion:**

Our study found that older patients had mixed feelings about changing their behaviour. The majority initially stated that dietary intake and physical activity were not a priority at older age. However, with knowledge that behaviour could improve health, patients also stated willingness to change, leading to a state of ambivalence. Healthcare professionals may consider motivational interviewing techniques to address this ambivalence.

**Supplementary Information:**

The online version contains supplementary material available at 10.1007/s41999-023-00774-1.

## Key summary points

**Aim:**

To explore barriers and facilitators regarding dietary intake and physical activity behaviour change in older patients undergoing transcatheter aortic valve implantation.

**Findings:**

Three following themes were identified as barriers: (1) low physical capability, (2) healthy dietary intake and physical activity are not a priority at an older age and (3) ingrained habits and preferences. Three themes were identified as facilitators: (1) knowledge that dietary intake and physical activity are important for maintaining health, (2) norms set by family, friends and caregivers and (3) support from the social environment.

**Message:**

Given the prevalent ambivalence among older cardiac patients towards behaviour change, healthcare professionals should address this mindset before implementing interventions to promote behaviour modification.

**Supplementary Information:**

The online version contains supplementary material available at 10.1007/s41999-023-00774-1.

## Introduction

With the development of minimally invasive techniques for cardiac procedures, the number of older patients who are eligible for elective cardiac procedures is steadily increasing [1]. For example, the number of older patients with severe aortic stenosis who qualify for transcatheter aortic valve implantation (TAVI) has grown significantly in recent years [1]. However, even after a successful procedure, patients who are at risk of malnutrition or have a low physical activity level are at increased risk of adverse outcomes, such as physiological decline and early mortality [2, 3].

Dietary intake and physical activity are important modifiable risk factors [4, 5, 6, 7, 8]. Recent studies show that a majority (up to 70%) of older patients scheduled for a cardiac procedure do not meet the physical activity guidelines, and up to 40% are at risk of malnutrition [5, 6]. Interventions to improve dietary intake and physical activity could lead to better clinical outcomes, as shown in several populations undergoing a cardiac procedure [9, 10, 11]. These interventions often included an increase in protein intake with the encouragement of protein-rich products or supplements. Change in physical activity behaviour often involve exercise during multiple sessions per week or encouragement to increase habitual physical activity [12]. To maintain health, patients should be motivated to eat at least 1.2 g/kg of protein per day in combination with a healthy diet [13]. This diet should be combined with at least 150 min of moderate-intensity physical activity per week to maintain functioning and health [14]. Despite the effectiveness of interventions on dietary intake and physical activity, there is evidence that adherence to behaviour change, especially in the older patient population, is often low [15].

According to the capability, opportunity and motivation behaviour model (COM-B). Change in behaviour is dependent on the physical and psychological capability of a person, the social and physical opportunity and the automatic and reflective motivation to perform behaviour change [16]. If one of these conditions is not met, diet and activity behaviour will not be changed [18]. Some conditions within the framework can also be used to facilitate behaviour change. The presence of a disease may be a factor that affects the capability, opportunity or motivation towards behaviour change [17]. It is unknown in older cardiac patients, such as the TAVI patient, which factors within the COM-B model lead to barriers or facilitators of behaviour change. This study aims to contribute to the understanding of barriers and facilitators of change in dietary intake and physical activity behaviour in older patients undergoing TAVI.

## Methods

We used a qualitative design with semi-structured interviews to explore the patients’ views. The consolidated criteria for reporting qualitative research (COREQ) checklist was used for reporting [18]. This qualitative study was nested within a larger project to improve dietary intake and physical activity in older cardiac patients. Our study is approved by the local medical ethics committee (METC, W19_317) and informed consent was obtained from all participants.

### Participants

We included patients with severe aortic valve stenosis planned for a transfemoral or transaortic TAVI from a university hospital that serves a large region. Patients were recruited by telephone after the heart team decided for the TAVI or one or three months after the procedure. We used purposeful sampling to collect the data of the heterogeneous group with the following characteristics: access route (transfemoral and transaortic), age (70–80 and > 80), sex (male and female) and New York heart association (NYHA) class before TAVI (I/II and III/IV). All patients were interviewed once during a period ranging from 2 months before to 3 months after the procedure. The only exclusion criterion was the inability to communicate in Dutch. The number of included participants was based on data saturation. After the expected saturation was reached, two additional interviews were conducted. When no new codes were added, data saturation was confirmed.

### Data collection

Data were collected using semi-structured interviews at the participants’ homes by DE (MSc, male) or CD (MSc, female). There was no strict time limit for the duration of the interviews, patients were informed that the interview would typically take around 1 hour. DE has a master’s degree in human movement sciences, and CD has a master’s degree in evidence-based healthcare and works as a senior nurse. The participants were interviewed individually. The interviewers were not directly involved in the care of the participants.

The researchers assumed that behaviour is determined by the capability, opportunity and motivation behaviour (COM-B) model [16]. In this model, capability is the psychological and physical capability of the person to perform a certain behaviour, opportunity is the physical and social environment that enables a certain behaviour, and motivation is the will of the person to perform a certain behaviour. The COM-B model has been shown to be helpful for obtaining a deeper exploration of barriers and facilitators that influence behaviour [16].

A semi-structured interview guide with open questions on the aim of this study was developed (Supplemental material, Table 1). The interview guide was checked for completeness and understanding from a patient perspective by a former patient working as a communication specialist at the heart centre. Depending on the answers given by participants, more in-depth questions were asked.

Data collection from usual care were used as baseline characteristics which were nutritional status (using the mini nutritional assessment—short form (MNA-SF)), age, sex, body mass index (BMI), living situation, TAVI access site and NYHA score before the TAVI,

### Analysis

All interviews were audio-recorded and transcribed verbatim, and checked for accuracy against the audio record. We did not seek feedback or corrections from participants. To identify the data saturation point, we conducted simultaneous data collection and analysis. The data were coded using MAXQDA 2022 software and analysed using Braun and Clarke’s six phases of thematic analysis [19]. The COM-B model was used as a framework under which themes were categorised [16]. In the first phase, the interviews were transcribed verbatim. Interviews were read several times to ensure the content was familiar to the researchers. In the second phase, the interviews were open-coded by one researcher (DE) and checked by another (CD). Any discrepancies were discussed until a consensus was reached. In the third phase, the open codes were sorted into themes under the COM-B framework. In the fourth phase, the themes were reviewed, discussed and checked against the original data until an agreement was reached. In the fifth phase, themes were further defined and named. Themes were classified as a barrier or facilitator and related to dietary intake, physical activity or both. In the final phase, the quotes were selected and translated for the research report, and findings were compared to the existing literature. To ensure trustworthiness, we recorded the iterative development process of data understanding. The records included all the ideas and thoughts of the researchers and were used to build understanding. Additionally, we consistently focused on the meaning of participants’ transcripts and their relation to the identified themes. Consequently, participants’ quotes are used to present the results.

## Results

A total of 13 interviews were conducted until data saturation was reached. The participants had an average age of 82 ± 6 years and 6 were females (Supplemental material, Table 2). Six themes were identified (Table 1). These themes were evenly distributed, with three being barriers and three being facilitators for healthy behaviour changes. All themes were related to both dietary intake and physical activity. Within the COM-B model two themes were related to capability, two to opportunity and two to motivation.

**Table 1:** Identified themes

	Themes	
Barriers	Lower physical capability	Capability
	Healthy dietary intake and physical activity are not a priority at an older age Ingrained habits and preferences	Motivation Motivation
Facilitators	Knowledge that dietary intake and physical activity are important for maintaining health	Capability
	Norms set by family, friends and caregivers Support and guidance from the social environment	Opportunity Opportunity

### Barriers

#### Lower physical capability

Lower physical capability was a common barrier for many patients. Factors such as lower appetite, fatigue, pain, and dizziness hindered their ability to improve their dietary intake and increase physical activity before or after their cardiac procedure.

P11: The dizziness restricts the amount of physical activity I can perform. I regret that because I am motivated to perform more activities.

P06: I had no appetite, I did not eat for weeks.

P05: I can’t be more physically active, because my arms and my legs are not functioning well.

P02: Currently, I have a decent dietary intake, but during the preprocedural period, I did not feel like eating. Although I needed it, which I understand, but I did not have the energy.

#### Healthy dietary intake and physical activity are not a priority at an older age

Another barrier was the lack of motivation to prioritize healthy dietary intake and physical activity at an older age. Many patients expressed a preference for a relaxed lifestyle without the need to exercise or focus on their dietary choices. Despite being aware of their poor dietary habits and low activity levels, they were not motivated to make changes.

P10: No, no, I am terribly inactive, I am not motivated to do activities requiring exercise, it is laziness, just laziness. It is easy like that, I don’t mind that I am not doing it. I am having fun without those activities.

P06: Yes, I became lazier, let me put it like that. But that is just what I want, I want to become lazier for me it is not necessary.

P04: Actually, what I like, I eat, but what I do not like, I don’t eat…At my age, let the good things come.

P13: But yes, I mean, building strength, I have done that years ago when I was much younger, but currently I do not care anymore. Then I think, for what?

#### Ingrained habits and preferences

Finally, ingrained habits and preferences were a barrier for patients. At an older age, patients had developed certain patterns of behaviour with a preferred diet and activity level, which were difficult to modify. These ingrained habits and preferences made it challenging for patients to make changes to their behaviour.

P04: Well, yes, you do it automatically, because of what I just told you. I have been doing that for years. I did not write down the date, but I cannot remember that I did not do it.

P01: Order and regularity, because that's what I'm used to.

P10: I have a set schedule that I live in, and I prefer that.

P03: I don’t want to adjust anymore. No, at the moment, it goes well, and I won't change anything.

### Facilitators

#### Knowledge that dietary intake and physical activity are important for maintaining health

When patients had knowledge about the importance of healthy dietary intake and physical activity for maintaining health, they stated they were willing to make changes. Even if they initially stated that it was not a priority, the maintenance of health could be a reason to adjust behaviour.

P011: Yes, I am willing to become more physically active to stay healthy.

P13: If it has to do with my health, then I'm open to it, of course, because who doesn't want to be healthy?!

P012: When it is good for body and soul, I would, of course, do it. Yes, I don’t want to die.

P04: I prefer salty food, I often think that my food is too bland. But now I pay attention to my salt intake. No more salt on the potatoes for instance. During cardiac rehabilitation we had a session about dietary intake, they had a whole explanation about healthy diets with among others salt intake, which was really helpful.

P04: You sometimes read something about healthy food. Recently, as an example. I read that it is extremely healthy to eat tuna. Since then I buy cans of tuna.

#### Norm set by family, friends, caregivers

Another facilitator was the influence of family and friends on setting a new norm for healthy behaviour. When patients were motivated to improve, a family member or caregiver could help them improve their dietary quality and physical activity levels by setting a new norm. However, the opposite was also true, as negative influences could hinder behaviour change.

P01: My oldest daughter graduated from the hotel school. She made a list years ago of what I should not eat, so I stick to that, more or less.

P06: The cardiologist also said, ‘Do you want to exercise? Well, I won't recommend it to you, because you're at an age when you don't have to walk so fast anymore’…I adhere to that.

P01: I was at my general practitioner, and he said you should stop smoking and beer drinking, because I drank a few beers every day. He said you have to stop both smoking and beer drinking. Then I said that's no problem, because I'll just stop with that yes…. I have stopped smoking and only occasionally drink a beer.

#### Support from the social environment

Finally, support from the social environment were crucial for facilitating healthy behaviour changes. Many patients mentioned that they were able to be more active and have healthy meals when they received support from family, friends, or caregivers. This support and guidance were essential for helping patients accommodate changes in their behaviour.

P08: I need somebody who walks along with me. I cannot walk for a long distance on my own. So that person needs to have time to build that up with me, so to speak.

P02: My son and daughter-in-law. Very often they come to cook a healthy meal for me.

P12: Yes, I have a sister, she takes care of me, she comes to me and goes walking with me, I really appreciate that.

P07: No, you really need good guidance. They have to contact you every week. Well then you hear how it goes. That is guidance.

## Discussion

This qualitative study aimed to explore barriers and facilitators of behaviour change in dietary intake and physical activity among older patients undergoing TAVI, with the COM-B model as a structure. We identified three barriers and three facilitators of behaviour change, which were all related to both dietary intake and physical activity.

According to the COM-B model, individuals must be capable, have the social and environmental opportunity and be motivated to perform a certain behaviour [20]. Our interviews revealed that older cardiac patients experience a barrier in motivation because they do not prioritize dietary intake and physical activity. This lack of priority often had to do with their desire to spend their remaining years without having to exercise or adhere to dietary restrictions. However, when patients were more aware of the importance of dietary intake and physical activity for maintaining or improving health, there was an ambivalence in their motivation to change behaviour. An earlier study in older patients showed similar findings and described that motivation for behaviour change is determined by a balance between quality of life (living according to preferences) and health benefits [21]. During periods of illness, patients are more aware of their behaviour and this has been shown to be a motivator to shift the balance towards a healthier lifestyle [17]. Therefore, the period before and shortly after a cardiac procedure can be an opportune moment for healthcare professionals to explore and resolve patients’ ambivalence using motivational interviewing techniques [22].

Another important motivational barrier to behaviour change was ingrained habits and preferences. At an older age, patients have developed preferred routines that they have performed for many years, sometimes even decades. Since patients have reached an older age with this routine, they are often convinced that their behaviour does not need any modification. This belief should be taken into account when older patients make changes in their behaviour. A systematic review of behaviour change showed that holding on to habits and preferences mainly applies to daily habits and is not a barrier to prescribed instructions [23]. Earlier research has shown that interventions focused on prescribed extra protein and two hours of weekly exercise for eight weeks can lead to a significant increase in muscle mass and muscle strength, without the need for a complete change in habitual behaviour [24]. Therefore, this approach of providing prescribed instructions on the most important aspects of dietary intake and physical activity might be a suitable way to overcome the barrier of habits and preferences.

To encourage healthy behaviour among motivated patients, it may be helpful to involve family, friends, and healthcare professionals by providing specific behavioural norms. Current guidelines for TAVI recommend that behavioural norms should be consistent with national and international guidelines and tailored to the patient's capabilities [25]. This is because the most optimal TAVI-specific norms have yet to be established. For dietary intake this means a minimal 1.2 g/kg protein per day and a better diet quality [13]. Diet quality is defined as a higher intake of nutrient-rich food groups (e.g. vegetables, fruit, whole grains) and low intake of nutrient-poor food groups (e.g. refined grains, sweets, alcohol). In the TAVI population, it is shown that a vast majority of the patients do not adhere to these guidelines, indicating that there is room for improvement [26]. For physical activity, a minimum of 150 min of moderate-intensity exercise and strength training two times a week is recommended [14, 25, 27]. Again, the majority of patients in this population do not meet these guidelines, indicating a need for improvement [5]. It is also important to note that even for patients unable to reach this amount of activity, any increase in activity can lead to health improvements [14, 27]. The social environment can facilitate improvement by providing support to patients [12].

Subsequently, patients should be supported in achieving their personal health goals. Many patients have limited physical capacity and may not be able to engage in healthy behaviours, such as cooking a healthy meal or going for a daily walk, despite their willing to do so. In these cases, the social environment can play a crucial role in facilitating healthier behaviours by providing support and recourses. For instance, family and friends can provide assistance by cooking healthy meals or participating in exercise programs together. Earlier studies have shown that involvement of the social environment can have a positive effect on sustainable improvement in health behaviour [28, 29, 30]. When patients live with a partner it is particularly important to include the partner in the behaviour change process, as partners often engage in activities and eat meals together. When the partner also participates in the behaviour change, the positive effect of involvement can be even greater [28, 31]. Therefore, interventions aimed at changing health behaviours should take the involvement of the social environment into account.

This study has several strengths. Firstly, the use of the COM-B framework allowed for a comprehensive understanding of patient behaviour and provided a direction for the study. The use of an established theory also enabled the researchers to build on existing knowledge and increased the information power of the study [32]. Moreover, the purposeful sampling technique enabled the inclusion of a diverse study population, providing a wide view of the barriers and facilitators experienced by TAVI patients. The study did not find any themes related to specific patient or procedural characteristics. Future studies could focus on experienced barriers and facilitators in specific subgroups of TAVI patients, for instance patients with malnutrition or high NYHA score. Furthermore, including patients before and after TAVI provided a comprehensive view of the issues associated with waiting for the procedure and recovering from the procedure. Because the interviews before and after did not yield distinct themes, they were analysed together. We were able to include multiple patients with a risk of malnutrition, which increased the transferability of the findings [6]. Some aspects of our study warrant consideration. Firstly, the population consisted of only TAVI patients in one geographic region, although this capital and rural environment represents several populations, this may limit the generalizability of the results to other older populations undergoing different cardiac procedures or patients in other regions. However the symptoms and other baseline characteristics of the included patients were comparable to other TAVI populations and other older and frail cardiac populations [33, 34, 35]. Furthermore, it is important to note that our center used guidelines in line with international guidelines for valve treatment, which means that similar patients were included for TAVI as in other regions and hospitals, which increases generalizability [36, 37]. Last, no objective data were available on participants' physical activity to present as a baseline characteristic. However, previous studies have shown that a majority of TAVI patients do not meet the minimal physical activity guidelines [38, 39].

In our study, we found that older patients undergoing TAVI had mixed feelings about changing their behaviour. Although many initially viewed dietary intake and physical activity as low priorities due to their age, they expressed a willingness to make changes when presented with information about the potential health benefits of these behaviours. This created a state of ambivalence among participants, who recognized the importance of behaviour change for their health but also felt unmotivated to make changes at their age. Healthcare professionals should address this ambivalence in patients and support those who are motivated to make changes in achieving their goals. This support can come from family, friends, and caregivers, who can set norms and provide encouragement for daily activity and a healthy diet. Additionally, low capability and ingrained habits and preferences may be important factors that need to be taken into account as barriers to behaviour change.

## Supplementary Information

Below is the link to the electronic supplementary material.

Supplementary file1 (DOCX 22 KB)

## Data Availability

The datasets generated and analysed during the current study are made available on the Figshare repository at 10.21942/uva.22360003.

## References

[1] Durko AP, Osnabrugge RL, van Mieghem NM, Milojevic M, Mylotte D, Nkomo VT (2018). Annual number of candidates for transcatheter aortic valve implantation per country: current estimates and future projections. Eur Heart J.

[2] Afilalo J, Lauck S, Kim DH, Lefèvre T, Piazza N, Lachapelle K (2017). Frailty in older adults undergoing aortic valve replacement: the FRAILTY-AVR study. J Am Coll Cardiol.

[3] Partridge JSL, Harari D, Dhesi JK (2012). Frailty in the older surgical patient: a review. Age Ageing.

[4] Goldfarb M, Lauck S, Webb JG, Asgar AW, Perrault LP, Piazza N (2018). Malnutrition and mortality in frail and non-frail older adults undergoing aortic valve replacement. Circulation.

[5] Sathananthan J, Lauck S, Piazza N, Martucci G, Kim DH, Popma JJ (2019). Habitual physical activity in older adults undergoing TAVR. JACC Cardiovasc Interv.

[6] Eichler S, Salzwedel A, Harnath A, Butter C, Wegscheider K, Chiorean M (2018). Nutrition and mobility predict all-cause mortality in patients 12 months after transcatheter aortic valve implantation. Clin Res Cardiol.

[7] Abeles A, Kwasnicki RM, Pettengell C, Murphy J, Darzi A (2017). The relationship between physical activity and post-operative length of hospital stay: a systematic review. Int J Surg.

[8] Lund K, Sibilitz KL, Berg SK, Thygesen LC, Taylor RS, Zwisler AD (2016). Physical activity increases survival after heart valve surgery. Heart.

[9] McCann M, Stamp N, Ngui A, Litton E (2019). Cardiac prehabilitation. J Cardiothorac Vasc Anesth.

[10] Baimas-George M, Watson M, Elhage S, Parala-Metz A, Vrochides D, Davis BR (2020). Prehabilitation in frail surgical patients: a systematic review. World J Surg.

[11] Marmelo F, Rocha V, Gonçalves D (2018). The impact of prehabilitation on post-surgical complications in patients undergoing non-urgent cardiovascular surgical intervention: systematic review and meta-analysis. Eur J Prev Cardiol.

[12] Dibben GO, Dalal HM, Taylor RS, Doherty P, Tang LH, Hillsdon M (2018). Cardiac rehabilitation and physical activity: systematic review and meta-analysis. Heart.

[13] Deutz NEP, Bauer JM, Barazzoni R, Biolo G, Boirie Y, Bosy-Westphal A (2014). Protein intake and exercise for optimal muscle function with aging: recommendations from the ESPEN Expert Group. Clin Nutr.

[14] Piercy KL, Troiano RP, Ballard RM, Carlson SA, Fulton JE, Galuska DA (2018). The physical activity guidelines for Americans. JAMA–J Am Med Assoc.

[15] Essery R, Geraghty AWA, Kirby S, Yardley L (2017). Predictors of adherence to home-based physical therapies : a systematic review. Disabil Rehabil.

[16] Michie S, van Stralen MM, West R (2011). The behaviour change wheel: a new method for characterising and designing behaviour change interventions. Implement Sci.

[17] Dijkstra SC, Neter JE, Brouwer IA, Huisman M, Visser M (2014). Motivations to eat healthily in older Dutch adults - a cross sectional study. Int J Behav Nutr PhyActivity.

[18] Tong A, Sainsbury P, Craig J (2007). Consolidated criteria for reporting qualitative research (COREQ): a 32-item checklist for interviews and focus groups. Int J Qual Health Care.

[19] Braun V, Clarke V (2006). Using thematic analysis in psychology. Qual Res Psychol.

[20] Barker F, Atkins L, de Lusignan S (2016). Applying the COM-B behaviour model and behaviour change wheel to develop an intervention to improve hearing-aid use in adult auditory rehabilitation. Int J Audiol.

[21] Jepma P, Snaterse M, du Puy S, Peters RJG, op Reimer WJMS (2021). Older patients’ perspectives toward lifestyle-related secondary cardiovascular prevention after a hospital admission—a qualitative study. Age Ageing.

[22] Rollnick S, Butler CC, Kinnersley P, Gregory J, Mash B (2010). Motivational interviewing. BMJ.

[23] Franco MR, Tong A, Howard K, Sherrington C, Ferreira PH, Pinto RZ (2015). Older people ’ s perspectives on participation in physical activity : a systematic review and thematic synthesis of qualitative literature. Br J Sports Med.

[24] de Liao C, Tsauo JY, Wu YT, Cheng CP, Chen HC, Huang YC (2018). Effects of protein supplementation combined with resistance exercise on body composition and physical function in older adults: A systematic review and meta-analysis. Nutrients.

[25] Chatrath N, Papadakis M (2022). Physical activity and exercise recommendations for patients with valvular heart disease. Heart.

[26] van Erck D, Tieland M, Adriaens NW, Weijs PJM, Scholte op Reimer WJM, Henriques JP (2022). GLIM-based malnutrition, protein intake and diet quality in preprocedural transcatheter aortic valve implantation (TAVI) patients. Clin Nutr.

[27] WHO (2020). Guidelines for physical activity and sedentary behaviour.

[28] Verweij L, Jørstad HT, Minneboo M, ter Riet G, Peters RJG, Scholte op Reimer WJM (2021). The influence of partners on successful lifestyle modification in patients with coronary artery disease. Int J Cardiol.

[29] Wood DA, Kotseva K, Connolly S, Jennings C, Mead A, Jones J (2008). Nurse-coordinated multidisciplinary, family-based cardiovascular disease prevention programme (EUROACTION) for patients with coronary heart disease and asymptomatic individuals at high risk of cardiovascular disease: a paired, cluster-randomised controlle. Lancet.

[30] Murray J, Craigs CL, Hill KM, Honey S, House A (2012). A systematic review of patient reported factors associated with uptake and completion of cardiovascular lifestyle behaviour change. BMC Cardiovasc Disord.

[31] Jackson SE, Steptoe A, Wardle J (2015). The influence of partner’s behavior on health behavior change: the english longitudinal study of ageing. JAMA Intern Med.

[32] Malterud K, Siersma VD, Guassora AD (2015). Sample size in qualitative interview studies: guided by information power. Qual Health Res.

[33] Jepma P, Verweij L, Buurman BM, Terbraak MS, Daliri S, Latour CHM (2021). The nurse-coordinated cardiac care bridge transitional care programme: a randomised clinical trial. Age Ageing.

[34] van Seben R, Reichardt LA, Aarden JJ, van der Schaaf M, van der Esch M, Engelbert RHH (2019). The course of geriatric syndromes in acutely hospitalized older adults: the hospital-ADL study. J Am Med Dir Assoc.

[35] Olsson K, Nilsson J, Hörnsten Å, Näslund U (2017). Patients’ self-reported function, symptoms and health-related quality of life before and 6 months after transcatheter aortic valve implantation and surgical aortic valve replacement. Eur J Cardiovasc Nurs.

[36] Otto CM, Kumbhani DJ, Alexander KP, Calhoon JH, Desai MY, Kaul S (2017). 2017 ACC expert consensus decision pathway for transcatheter aortic valve replacement in the management of adults with aortic stenosis. J Am Coll Cardiol.

[37] Otto CM, Nishimura RA, Bonow RO, Carabello BA, Rwin JP, Gentile F (2021). 2020 ACC/AHA guideline for the management of patients with valvular heart disease: a report of the American college of cardiology/American heart association joint committee on clinical practice guidelines. Circulation.

[38] van Erck D, Dolman CD, Scholte op Reimer WJM, Henriques JP, Weijs PJM, Delewi R (2022). The trajectory of nutritional status and physical activity before and after transcatheter aortic valve implantation. Nutrients.

[39] Haum M, Humpfer F, Steffen J, Fischer J, Stocker T, Sadoni S (2023). Quantification of physical activity with activity tracking after transfemoral aortic valve replacement (TAVR). Eur Heart J.

