# Supplementary material for: Exploring barriers and facilitators of behavioural changes in dietary intake and physical activity: a qualitative study in older adults undergoing transcatheter aortic valve implantation
Source: Eur Geriatr Med. 2023 Apr 1;14(3):503–10. doi: 10.1007/s41999-023-00774-1 (PMC10261232; doi:10.1007/s41999-023-00774-1)
Supplement: Supplementary file 1 — Supplementary file1 (DOCX 22 KB) [file 41999_2023_774_MOESM1_ESM.docx]

*Supplementary Table 1: Interview guide*

| Physical Activity | Dietary intake |
| --- | --- |
| Can you tell something about your current physical activity pattern? | Can you tell something about your current dietary intake pattern? |
| Has your activity pattern changed in the last year? | Has your dietary pattern changed in the last year? |
| Has the aortic stenosis or TAVI changed your activity pattern? If yes, why and how | Has the aortic stenosis or TAVI changed your dietary intake? If yes, why and how |
| Do you experience barriers or facilitators to become more physically active? | Do you experience barriers or facilitators to eat more healthy? |
| Which factors in your environment influence your physical activity pattern? | Which factors in your environment influence your dietary intake? |
| Which factors in your social environment influence your physical activity pattern? | Which factors in your social environment influence your dietary intake? |
| Are you motivated to change your activity pattern? and why or why not? | Are you motivated to change your dietary pattern? and why? |

|  | **Age** | **Sex** | **BMI, kg/m^2^** | **Living situation** | **MNA-SF** | **Nutrition status** | **Access site** | **NYHA  before TAVI** |
| --- | --- | --- | --- | --- | --- | --- | --- | --- |
| P01 | 85 | M | 30.2 | Alone | 14 | Normal | TF | I |
| P02 | 90 | F | 22.9 | Alone | 7 | Malnourished | TAO | II |
| P03 | 79 | F | 32.9 | Partner | 14 | Normal | TF | III |
| P04 | 90 | M | 26.3 | Partner | 14 | Normal | TAO | I |
| P05 | 73 | F | 24.4 | Alone | 11 | At risk | TF | IV |
| P06 | 78 | M | 25.6 | Partner | 7 | Malnourished | TF | III |
| P07 | 77 | M | 33.1 | Alone | 10 | At risk | TF | II |
| P08 | 82 | F | 22.1 | Alone | 11 | At risk | TAO | III |
| P09 | 86 | M | 25.7 | Alone | 14 | Normal | TF | I |
| P10 | 90 | M | 33.8 | Alone | 14 | Normal | TF | III |
| P11 | 72 | F | 30.8 | Alone | 14 | Normal | TF | I |
| P12 | 83 | F | 25.6 | Partner | 8 | At risk | TF | II |
| P13 | 79 | M | 26.0 | Partner | 10 | At risk | TAO | I |

*Supplementary Table 2: Patient characteristics*

BMI: body mass index, MNA-SF: mini nutritional assessment – short form, TF: transfemoral, TAO: transaortic, NYHA: New York Heart Association

*Supplementary Table 3: Labels found leading to themes*

| **Capability** | |
| --- | --- |
| **Lower physical capability** | **Knowledge that dietary intake and physical activity are important for maintaining health** |
| Altered taste and appetite | Learning new information about healthy diet or activity level from media |
| Physical ageing | Common knowledge about healthy behaviour and influence on health |
| Symptoms of aortic stenosis | Knowledge gain on healthy behaviour from friends, and family |
| Comorbidities |  |
| Poor physical condition |  |
|  | |
| **Opportunity** | |
| **norm set by social environment** | **Social Support by family and friends** |
| Influence from partner, family or friends on lifestyle | Family support with daily activities (groceries, cooking, going for walks) |
| Lifestyle recommendations by caregiver | Eating or exercising together |
| Conformation to the environment |  |
|  | |
| **Motivation** | |
| **Healthy dietary intake and physical activity are not a priority at an older age** | **Ingrained habits and preferences** |
| Prioritize comfort in the final years of life | Routine in consumed meals and daily activities |
| Belief that lifestyle behaviour is not important for health at an older age | Eating according to preferences |
|  | Doing preferred activities |
|  | Influence of past habits on current activities and dietary intake |
